# β-Cyclodextrin-Based Nanosponges Functionalized with Drugs and Gold Nanoparticles

**DOI:** 10.3390/pharmaceutics13040513

**Published:** 2021-04-08

**Authors:** Isabel Asela, Orlando Donoso-González, Nicolás Yutronic, Rodrigo Sierpe

**Affiliations:** 1 Laboratorio de Nanoquímica y Química Supramolecular, Departamento de Química, Facultad de Ciencias, Universidad de Chile, Las Palmeras 3425, Ñuñoa, 7800003 Santiago, Chile; i.asela.m@gmail.com (I.A.); orlando.donoso@ug.uchile.cl (O.D.-G.); 2 Laboratorio de Nanobiotecnología y Nanotoxicología, Departamento de Química Farmacológica y Toxicológica, Facultad de Ciencias Químicas y Farmacéuticas, Universidad de Chile, Santos Dumont 964, Independencia, 8380000 Santiago, Chile; 3 Laboratorio de Biosensores, Departamento de Química Farmacológica y Toxicológica, Facultad de Ciencias Químicas y Farmacéuticas, Universidad de Chile, Santos Dumont 964, Independencia, 8380000 Santiago, Chile

**Keywords:** β-cyclodextrin-based nanosponge, phenylethylamine, 2-amino-4-(4-chlorophenyl)thiazole (AT), gold nanoparticles, carrier of therapeutic agents

## Abstract

Drugs are widely used as therapeutic agents; however, they may present some limitations. To overcome some of the therapeutic disadvantages of drugs, the use of β-cyclodextrin-based nanosponges (βCDNS) constitutes a promising strategy. βCDNS are matrices that contain multiple hydrophobic cavities, increasing the loading capacity, association, and stability of the included drugs. On the other hand, gold nanoparticles (AuNPs) are also used as therapeutic and diagnostic agents due to their unique properties and high chemical reactivity. In this work, we developed a new nanomaterial based on βCDNS and two therapeutic agents, drugs and AuNPs. First, the drugs phenylethylamine (PhEA) and 2-amino-4-(4-chlorophenyl)-thiazole (AT) were loaded on βCDNS. Later, the βCDNS–drug supramolecular complexes were functionalized with AuNPs, forming the βCDNS–PhEA–AuNP and βCDNS–AT–AuNP systems. The success of the formation of βCDNS and the loading of PhEA, AT, and AuNPs was demonstrated using different characterization techniques. The loading capacities of PhEA and AT in βCDNS were 90% and 150%, respectively, which is eight times higher than that with native βCD. The functional groups SH and NH_2_ of the drugs remained exposed and allowed the stabilization of the AuNPs, 85% of which were immobilized. These unique systems can be versatile materials with an efficient loading capacity for potential applications in the transport of therapeutic agents.

## 1. Introduction

β-cyclodextrin (βCD) is a cyclic oligosaccharide approved by the FDA (Food and Drug Administration) that has been widely used as a pharmaceutical excipient in food products, textiles, cosmetics, and medical products [1]. In modern drug delivery investigations, βCD has been used as a host molecule for the preparation of drug carrier systems in diverse forms, such as vesicles, hydrogels, micelles, metal–organic systems, and nanoparticles [2,3,4,5,6]. Structural modifications of native βCD have been shown to increase its inclusion capacity and solubility and have allowed bioapplications of a large number of guest biomolecules [7,8,9]. An innovative modification to βCD recently studied was the synthesis of a polymeric cross-linked network, forming a highly porous and branched matrix of nanometric dimensions called the β-cyclodextrin-based nanosponge (βCDNS) [10,11]. This nanostructure contains multiple lipophilic cavities and carbonate bridges, leading to a network of hydrophilic channels [12], which allows βCDNS to serve as a polymeric conjugate, increasing the loading capacity, association, and stability of the included drugs [7,13,14,15,16,17,18,19]. Notably, a high loading capacity is a characteristic feature of βCDNS since they can interact with different molecules of suitable dimensions, using either the cavities of βCD or the multiple pores generated in the crosslinking [7,11,12]. Due to the above, studies on βCDNS applied to drug administration have been reported.

Drugs are some of the most widely used therapeutic agents; however, they may present some limitations, such as early instability, poor aqueous solubility, and low bioavailability. Therefore, strategies for the inclusion of drugs in matrices of native or modified βCD have been an excellent alternative for solving these disadvantages. In this work, the loading of the drugs phenylethylamine (PhEA) and 2-amino-4-(4-chlorophenyl)-thiazole (AT) on βCDNS was studied, which led to formation of two new systems: βCDNS–PhEA and βCDNS–AT. PhEA is a psychoactive stimulant that is used as an antidepressant without inducing tolerance; however, it is rapidly metabolized in organisms by the MAO-B enzyme, making it difficult to reach the site of action [20,21]. AT is a thiazole derivative that is currently used as an antimicrobial and anti-inflammatory agent but is rapidly degraded and has a poor aqueous solubility [22,23,24]. Our group previously studied complex formation between native βCD and these drugs. An increase was reported in the aqueous solubility and stability of PhEA over time due to its inclusion; in addition, the drug was released from the βCD–PhEA complex using laser irradiation and gold nanoparticles (AuNPs) present in the medium [25]. The inclusion of AT in βCD increased its aqueous solubility, allowing the application of higher doses in in vitro studies of permeability and antibacterial activity. Finally, it was demonstrated that the βCD–AT complex maintained its antibacterial activity against six strains of clinical relevance [26]. In this sense, the incorporation of βCDNS could show novel results, increasing the loading capacity or solubility, among other advantages [7,10,11,16]. Notably, AuNPs could also be added as a remarkable second therapeutic agent.

AuNPs have been widely employed in nanobiotechnology due to their unique properties, which allow them to be incorporated into new nanomaterials [27]. The main characteristics of AuNPs include their optoelectronic properties, as surface plasmon resonance (SPR), which are related to their shape, size, and large surface-to-volume ratio; besides its excellent biocompatibility and low toxicity [28]. The chemical reactivity of the surface atoms of AuNPs allows their functionalization and assembly with various chemical species, enabling their application in chemical and biological sensing, imaging, therapeutics, detection and diagnostics, biolabeling, and drug delivery [29,30,31,32,33,34,35]. Notably, AuNPs have been used as therapeutic and diagnostic agents, even in hard-to-reach places, such as the brain, since they can cross the blood–brain barrier [36,37,38,39,40]. Due to their photothermal properties, AuNPs can release compounds that are attached or close to its surface, such as drugs, at specific sites of action in a controlled manner due to the generation of thermal energy when excited by a laser specifically tuned to the SPR frequency [25,41,42]. Furthermore, AuNPs can accumulate passively in sites with an immature vasculature and with extensive fenestrations, such as tumor tissues, or in injured sites where an immune response develops. This is called the enhanced permeability and retention effect (EPR effect) [43,44,45]. It has been shown that the EPR effect combined with a longer blood circulation time of some types of nanoparticles can increase drug concentrations in tumors by 10 to 100 times compared to the use of free drugs [46]. In recent years, a series of complexes based on βCD have allowed the stabilization of AuNPs, building systems with promising applications in biological and chemical areas [25,26,47,48,49,50,51,52,53]. Therefore, if properly designed, βCDNS loaded with drugs and AuNPs could be used as new systems with biomedical applications, acting synergistically in nanotherapy.

In this work, we propose the development of a new system based on βCDNS for the transport of two therapeutic agents, drugs and AuNPs. For this, inclusions of PhEA and AT were studied separately. Later, the complexes were functionalized with AuNPs, forming the βCDNS–PhEA–AuNP and βCDNS–AT–AuNP systems. We believe that these unique systems, based on βCDNS, drugs, and AuNPs, can be versatile materials with potential applications in the therapy and diagnosis of diseases.

## 2. Materials and Methods

### 2.1. Material

Anhydrous βCD (C_42_H_70_O_35_) ≥ 97%, 1134.98 g/mol; diphenylcarbonate (DPC, C_6_H_5_O)_2_CO) 99%, 214.22 g/mol; PhEA (C_8_H_11_N) ≥ 99.5%, 121.18 g/mol, density (δ): 0.962 g/mL; AT (C_9_H_7_ClN_2_S) ≥ 98%, 210.68 g/mol; sodium hydroxide (NaOH) ≥ 97%, 40.00 g/mol; tetrachloroauric acid (HAuCl_4_) ≥ 99.9%, 393.83 g/mol; and sodium citrate (Na_3_C_6_H_5_O_7_) ≥ 99%, 294.10 g/mol were provided by Merck (Merck, Darmstadt, Germany). Ultrapure water (18 MWcm^−1^) was obtained from a Milli-Q water system (Synergy UV equipment, Merck, Darmstadt, Germany).

### 2.2. Synthesis of β-Cyclodextrin Nanosponges

For βCDNS synthesis, anhydrous βCD and DPC were used as precursors. Synthesis was carried out by adapting Patel’s protocol [54]. βCD (0.189 g) and 0.143 g of DPC were mixed in solid state at a 1:4 molar ratio βCD:DPC. A round-bottom flask with the mixture was heated inside an oil bath on a heating plate, with constant stirring for 5 h at 100 °C, observing its melting. The solid mixture obtained was ground in a mortar, washed with distilled water, and filtered under vacuum. The product was washed in a Soxhlet apparatus with acetone for 24 h, to remove phenol by-product. Later, it was moistened with water and dried for 2 h in a vacuum system using a Buchner funnel connected to a Kitasato flask to remove trace βCD. Finally, the product was dried for 72 h at 65 °C and stored.

### 2.3. β-Cyclodextrin Nanosponges Loading with Drugs

To load βCDNS with PhEA and AT, the saturated solutions method [55] was used with minor modifications. βCDNS were dispersed in a NaOH 0.1 M solution at room temperature, while the drugs were dissolved in ethanol. The solutions were mixed under constant agitation for 15 min and then left without agitation for 24 h. The resulting solution was centrifuged, and the supernatant was lyophilized and reserved [56,57]. The loading capacity of the βCDNS–PhEA and βCDNS–AT systems was calculated from the weights of βCD and drugs obtained using Equation (1) [58].

(1) $$\mathrm{Loading}\;\mathrm{capacity}=\;\frac{\mathrm{Weight}\;\mathrm{of}\;\mathrm{drug}\;\mathrm{in}\;\beta \mathrm{CDNS}}{\mathrm{Weight}\;\mathrm{of}\;\beta \mathrm{CD}\;\mathrm{in}\;\beta \mathrm{CDNS}}\;\times \;100$$

### 2.4. Association Constant, K_a_

For both drugs, studies were performed following the Higuchi and Connors method [59]. First, known concentrations (C) of each drug were measured by UV-Vis. From the A_max_ vs. C graph, the slope corresponded to the ε of each drug. Then, the βCDNS concentration versus the loaded drug concentration (calculated by Beer–Lambert law) was plotted. The value of the slope of the graphs related the amount of βCDNS added to the amount of solubilized drug, indicating the degree of solubilization. Degree of solubilization was used to calculate the association constant (K_a_) and complexation efficiency of each system using Equations (2) and (3), respectively.

(2) $$K_{a}{}_{\left(1:1\right)}\;=\;\frac{\mathrm{Degree}\;\mathrm{of}\;\mathrm{solubilization}}{\left[C_{o}\right]\left(1-\mathrm{Degree}\;\mathrm{of}\;\mathrm{solubilization}\right)}$$

(3) $$\mathrm{Complexation}\;\mathrm{efficiency}=K_{a}{}_{\left(1:1\right)}\left[C_{o}\right]\;=\;\frac{\mathrm{Degree}\;\mathrm{of}\;\mathrm{solubilization}}{\left(1-\mathrm{Degree}\;\mathrm{of}\;\mathrm{solubilization}\right)}$$

[C_o_] corresponds to the concentration of the free drug in the absence of βCDNS.

### 2.5. Synthesis of Gold Nanoparticles and Their Immobilization on β-Cyclodextrin Nanosponges–Drug Systems

Synthesis of AuNPs was performed using the Turkevich method [60]. A reflux system on a round-bottom flask (with three necks) was mounted by placing a thermometer, a condenser, and a rubber stopper on each neck. Here, 0.474 mL of HAuCl_4_ was added with 18 mL of water. Sodium citrate (22.8 mg) was dissolved in 2.0 mL of water and heated at 60 °C for approximately 5 min. When aqueous solution of HAuCl_4_ was refluxed and the gas–liquid equilibrium stabilized at a temperature of 186 °C, the citrate solution (at 60 °C) was added through the neck with the stopper. The reflux was continued under constant agitation (6× *g*) for 30 min until a deep red solution was obtained. Later, the solution was cooled slowly to room temperature. The obtained AuNPs were filtered, diluted, set to pH 8.8 using an NaOH solution, and stored at 4 °C.

Immobilization was carried out via solubilization of the βCDNS–drug supramolecular complexes in an alkaline environment of AuNPs, setting the pH to 8.8 using NaOH. These mixtures formed homogeneous colloidal solutions that were centrifuged to decant only the βCDNS–drug systems interacting with AuNPs. Once the systems βCDNS–drug–AuNP were separated from the supernatant, they were resuspended in a new aqueous solution, forming the systems βCDNS–AT–AuNP and βCDNS–PhEA–AuNP. The concentration of AuNPs was calculated using UV-Vis spectroscopy. The molar extinction coefficient was obtained from the literature [61,62], and it was applied together with the Beer–Lambert equation.

### 2.6. Analysis by Nuclear Magnetic Resonance of Protons, ^1^H-NMR

All the samples were dissolved in deuterated dimethylsulfoxide (DMSO)-d_6_.

### 2.7. Preparation of Samples for Studies by Scanning and Transmission Electron Microscopy, SEM and TEM 

For SEM studies, the βCD and βCDNS samples were prepared directly depositing the solid material onto carbon tape, then a gold coating was applied using magneton sputtering (pressure 0.5 mbar, Ar atmosphere, current 25 mA over 15 s). βCDNS–drug–AuNP samples were prepared by dropping aliquots on carbon tape, allowing them to dry overnight. The AuNPs immobilized on βCDNS–drug systems allowed the conductivity of these samples.

For TEM studies, the βCD and βCDNS samples were dissolved in ethanol (20% *v*/*v*), then mixed, sonicated, and dripped onto a copper grid with a continuous Formvar film. The βCDNS–drug samples were dissolved in ethanol (20% *v*/*v*), then mixed, sonicated, and dripped onto a holey carbon grid. Finally, all these samples were stained with phosphotungstic acid. The AuNPs samples were deposited directly on the grid with a continuous Formvar film.

### 2.8. Preparation of the Samples for Studies by Dynamic Light Scattering (DLS) and ζ Potential

βCDNS and βCDNS–drugs were redispersed to measurements. To determine the size distribution of the samples, the results were retrieved from the intensity distribution values using the cumulant method. The measurement conditions were set for organic βCD-based samples (refraction index: 1.49 and k: 0).

AuNPs and AuNPs with βCDNS–drug were diluted 10× for measurements. Sonication and filtration were performed through a 0.45 μm filter. To determine the size distribution of AuNPs on the samples, the results were retrieved from the intensity distribution values using the cumulant method. On the other hand, the Smoluchowski approximation was used to calculate the ζ potentials from the measured electrophoretic mobility. The measurement conditions were set for colloidal gold samples (refraction index: 1.33 and k: 0.20).

### 2.9. Equipment Used for Characterization of the Samples

#### 2.9.1. Nuclear Magnetic Resonance of Protons, ^1^H-NMR

^1^H-NMR characterizations of the βCDNS, PhEA, AT, and βCDNS–drug samples were performed in a Bruker Advance 400 MHz instrument (Bruker, Billerica, MA, USA) at 30 °C using TMS as an internal reference. The MestreNova program was used for data processing.

#### 2.9.2. Infrared Spectroscopy, IR

The analyses were performed on a Jasco FT/IR-4600 instrument (Jasco, Easton, PA, USA). Spectral resolution: 1 cm^−1^, number of scans: 4. CO_2_ and H_2_O correction through the software of the equipment was made. Baseline correction of KBr was performed.

#### 2.9.3. Thermogravimetric Analysis, TGA

Analyses were performed on Perkin-Elmer model 4000 equipment (Perkin-Elmer, Waltham, MA, USA) over a temperature range from 0 °C to 800 °C with a rate of 10 °C/min under an air atmosphere with a flow of 20 mL/min.

#### 2.9.4. Scanning and Transmission Electron Microscopy, SEM and TEM

For both characterizations, Inspect F50 HR-SEM instrument (Fei Company, Hillsboro, OR, USA) was used. For the scanning electron microscopy (SEM) images, an Everhart-Thornley detector was used, while for the transmission electron microscopy (TEM) images, the detector was scanning transmission electron microscope (STEM). An acceleration voltage of 10.0 kV, pressure of 9.71 × 10^−8^ Pa, and observation magnitudes of 16,000× and 100,000× were used.

#### 2.9.5. UV-Visible Spectrophotometry

A Shimadzu UV-2450 instrument (Shimadzu, Kyoto, Japan) was employed to obtain the absorbance spectra. Measurements were made in 1.0 cm diameter quartz cuvettes between 200 and 800 nm using water at pH 8.8 as the reference. The UVProve program, version 1.10, was used for data processing.

#### 2.9.6. Dynamic Light Scattering (DLS) and ζ Potential

The samples were measured on a Malvern Zetasizer Nano ZS instrument (Malvern, Malvern, UK).

#### 2.9.7. Lyophilization of the Samples

BenchTop Pro, Omnitronic team equipment, SP Scientific (Omnitronic team, Gardiner, NY, USA) was used.

For data processing and graphic design, OriginPro 8.0 software (OriginLab, Northampton, MA, USA) was used.

#### 2.9.8. Metallization of the Samples

PELCO SC-6 magnetron sputtering (PELCO, Fresno, CA, USA) was used. A gold foil was placed inside the vacuum chamber at 0.5 mbar, under inert atmosphere of argon. To begin the process, a current of 25 mA was used over 15 s to ionize the gas, hitting the metal foil and releasing Au atoms. These Au atoms were deposited over the βCD and βCDNS systems.

## 3. Results and Discussion

### 3.1. Synthesis and Characterization of β-Cyclodextrin-Based Nanosponges

**Figure A1:**
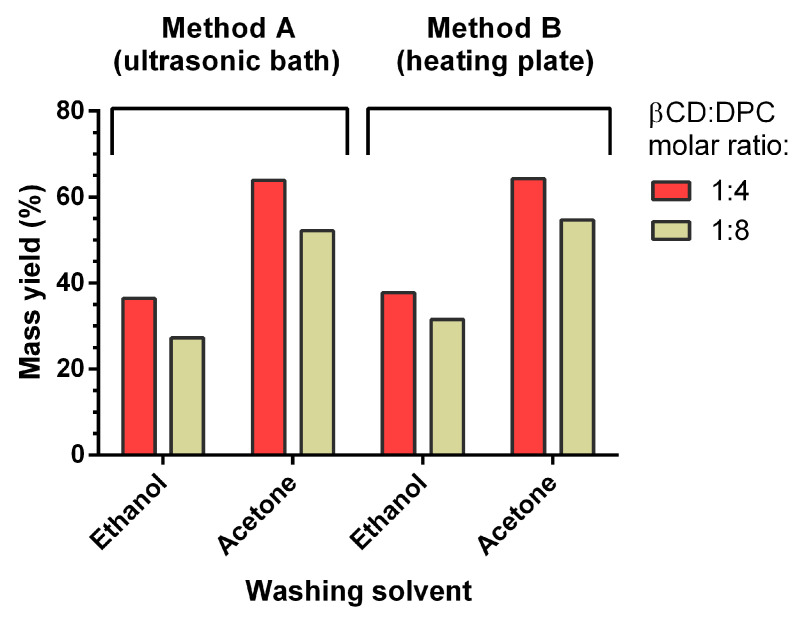
Mass yield (in percentage) of βCDNS versus the washing solvent, heating method and βCD:DPC molar ratio.

Different synthesis routes have been reported for βCDNS formation, and they use ultrasonic baths; heating plates; solvents, such as ethanol or acetone for the washing stages; and even different molar ratios of βCD and DPC [7,10]. For this reason, different methodologies were evaluated to optimize the synthesis of βCDNS, eliminate byproducts, and increase yield. For the ultrasonic bath (A) and heating plate (B) methods, the use of acetone and a 1:4 molar ratio showed yields greater than 60%, as shown in Figure A1 (Appendix A). Considering the reproducibility of the synthesis and the lower amount of generated byproducts exhibited by method B relative to method A, method B with a heating plate was selected.

**Figure 1:**
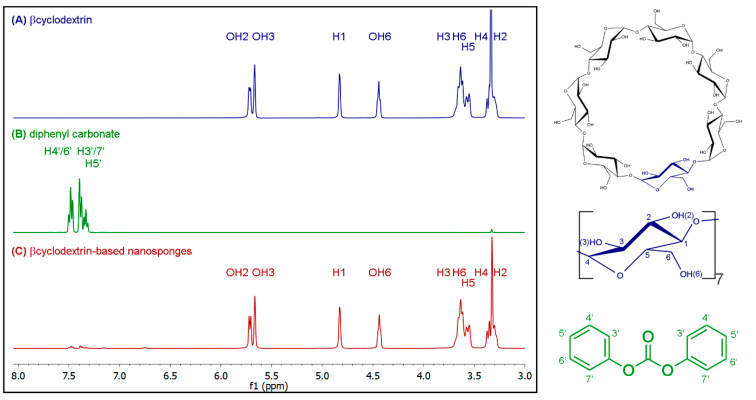
^1^H-NMR spectra of (**A**) native β-cyclodextrin (βCD), (**B**) diphenylcarbonate (DPC), and (**C**) β-cyclodextrin-based nanosponge (βCDNS) synthesized (**left**) together with the molecular structures and the assignments of the protons of βCD and DPC (**right**).

βCDNS formation was confirmed using ^1^H-NMR. The technique allowed us to compare the chemical shifts of the signals for βCD protons in βCDNS and in native βCD. Figure 1 shows the spectra of (A) βCD, (B) DPC, and (C) βCDNS with a scheme showing the proton assignments for βCD and DPC. Table 1 shows the proton assignment for βCD and their respective chemical shifts and integrations in the ^1^H-NMR spectra. The shifts of the signals are due to the change in the chemical environment of the βCD matrices when they are linked to form βCDNS. Notably, the greatest changes were observed in the integration delta (Δ∫) of the hydroxyl groups, because they react with DPC to form linkers between βCD matrices, strongly suggesting βCDNS formation.

**Figure 2:**
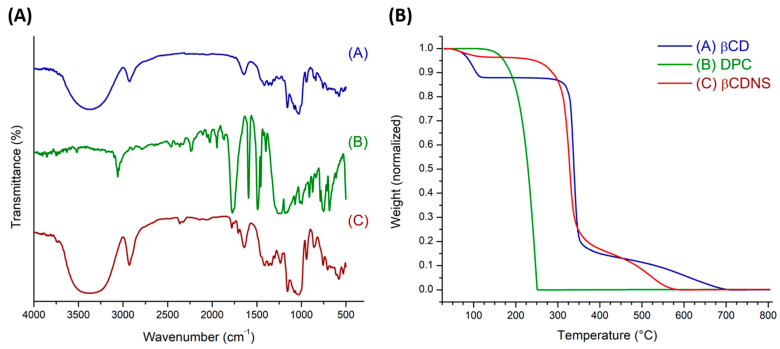
(**A**) FT-IR spectra of (A) βCD, (B) DPC, and (C) βCDNS; (**B**) normalized thermograms of (A) βCD, (B) DPC, and (C) βCDNS.

βCDNS formation was also characterized using IR vibrational spectroscopy. Commonly, this study focuses on comparing the signals of native βCD and βCD forming nanosponges and recognizing the vibration signal of the carbonyl group, which is an indicator of βCD crosslinking. Figure 2A shows the IR spectra of (A) βCD, (B) DPC, and (C) βCDNS. Characteristic peaks of βCD are observed at 3363 cm^−1^ (O-H alcohol stretching), 2924 cm^−1^ (C-H stretching), 1417 cm^−1^, 1368 cm^−1^, 1157 cm^−1^ (O-H bending), 1080 cm^−1^, and 1029 cm^−1^ (C-O stretching). These data are consistent with literature data [63,64,65]. For βCDNS, the characteristic peaks are located mostly in the same regions observed for βCD, but with shifts or variations in intensity due to changes in the chemical environment. These were observed at 3366 cm^−1^ (O-H alcohol stretching), 2928 cm^−1^ (C-H stretching), 1645 cm^−1^ (C=O stretching), 1367, 1234, and 1155 cm^−1^ (O-H bending), and 1079 cm^−1^ and 1030 cm^−1^ (C-O stretching). Notably, the appearance of peaks at 1783, 1715, and 1235 cm^−1^ derived from signals present in DPC confirm the crosslinking of βCD forming nanosponges. The peak at 1760 cm^−1^ (C=O stretching) of DPC is masked by a peak in the βCDNS spectrum.

**Figure A2:**
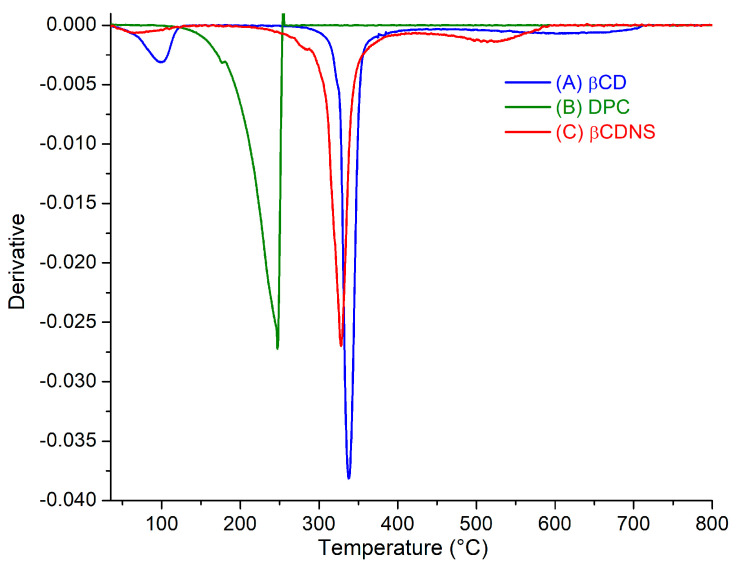
TGA derivative curves of (A) βCD, (B) DPC and (C) βCD.

Thermogravimetry was performed to analyze and confirm the formation of βCDNS, differentiating it from its precursors through changes in their thermal decomposition, as is typically observed in the synthesis of polymeric materials [66]. Figure 2B shows thermograms of (A) βCD, (B) DPC, and (C) βCDNS. The loss of hydration water was observed in the first decomposition at temperatures up to 100 °C, with the percentage of mass loss being 11.5% for βCD and 2.7% for βCDNS of the total mass samples. Decomposition of 100% of the mass of DPC was observed in the range 130 to 250 °C. A second range of decomposition in βCD was observed between 300 and 350 °C, corresponding to a loss of 71% of the sample mass. For βCDNS, this second range was between 210 and 350 °C, consuming 70% of the total mass. The decrease in the temperature at the beginning of thermal degradation suggests that DPC, a crosslinker molecule, binds to the primary OH groups of βCDs, forming the nanopolymer through carbonyl groups. Changes in the peaks of the TGA curves (see Figure A2 in Appendix A) from 337 °C (βCD) to 327 (βCDNS) are typically observed in the formation of polymeric materials due to changes in chemical structure [67,68,69]. Finally, the oxidation interval for βCD ranged from 350 to 700 °C, encompassing 17.5% of the mass. However, βCDNS oxidation ranges from 350 to 580 °C, encompassing 27.3% of the mass. This also suggests modifications in the reactive structure of the polymer relative to native βCD.

To explain the change in the beginning of the range of thermal degradation for βCDNS, the average between the beginning temperatures for βCD and DPC, which were 300 and 130 °C, respectively, was evaluated. The calculated average temperature was 215 °C, which coincided with the value of the beginning of thermal degradation observed in the βCDNS thermogram, fulfilling the “eutectic mixture” criterion [70]. In addition, the high value of the degradation interval for βCDNS supports its thermal stability.

**Figure 3:**
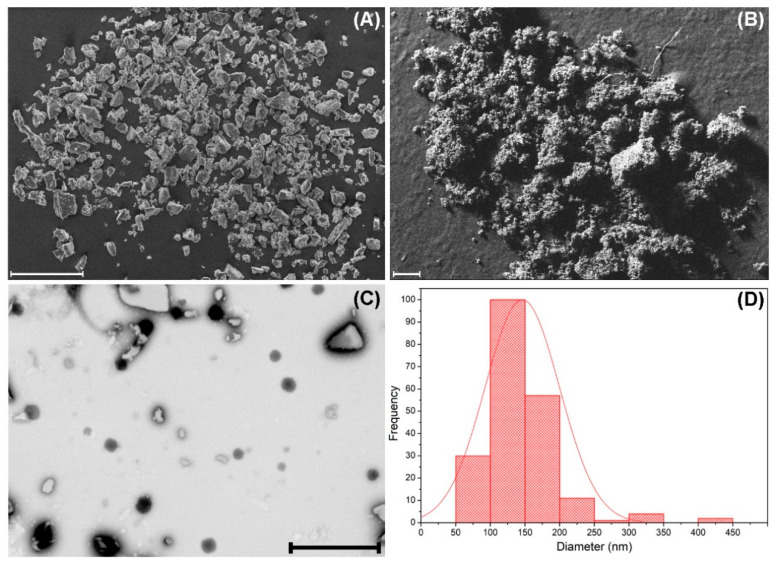
SEM micrographs of (**A**) βCD and (**B**) βCDNS. (**C**) transmission electron microscopy (TEM) micrograph of βCDNS and (**D**) the size distribution observed in TEM micrographs of βCDNS. Scale bar for figure (**A**) and (**B**) is 200 µm; scale bar for figure (**C**) is 1000 nm.

**Figure A3:**
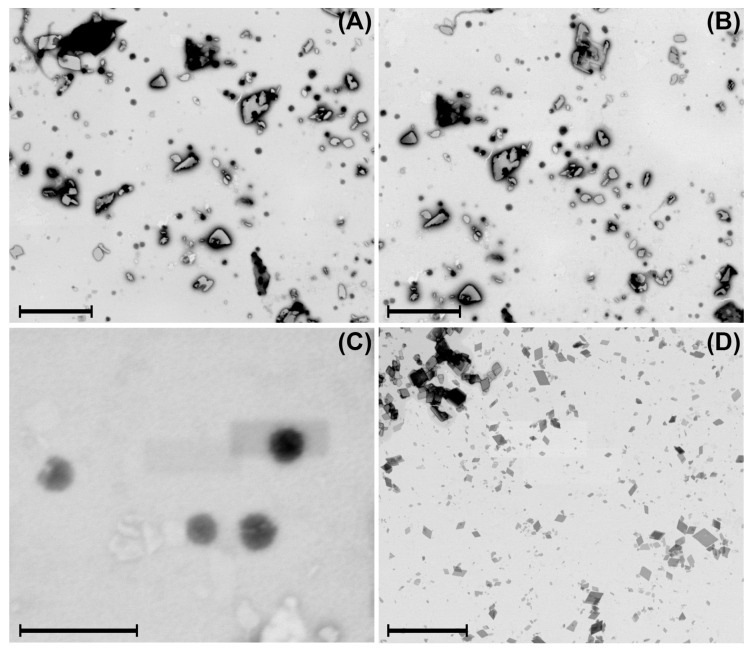
TEM micrographs of: (**A**,**B**) βCDNS with bar scales of 2000 nm; (**C**) βCDNS with bar scale of 400 nm; and (**D**) native βCD with bar scale of 4000 nm. Acceleration voltage used: 10.0 kV.

To obtain information on the morphology and size of βCDNS, the material was characterized using electron microscopy techniques and DLS. Figure 3 shows micrographs obtained by FE-SEM of native βCD (Figure 3A) and βCDNS (Figure 3B), directly revealing the morphological differences between both. βCD has irregular crystalline structures, while βCDNS has a characteristic porous appearance. TEM images were obtained to determine the average diameter of βCDNS, which were previously dispersed by sonication. Figure 3C,D shows the βCDNS and the resulting histogram, respectively. The average diameter, obtained from the count of more than 450 nanoparticles seen in various TEM images, was 146 ± 54 nm (see more images in Figure A3 in Appendix A). The staining of the βCDNS sample revealed some βCD crystals, which was verified by obtaining TEM images of native βCD with the same dispersion and staining protocol described for βCDNS (see Figure A3 in Appendix A). In addition, a hydrodynamic diameter of 133.9 ± 66.9 nm was found for βCDNS using DLS. These size data are concordant and strongly suggest the nanometric dimensions of the system studied (see more details in Appendix C).

### 3.2. Loading of β-Cyclodextrin-Based Nanosponges with Drugs

The βCDNS obtained was loaded with two drugs separately, forming the βCDNS–PhEA and βCDNS–AT systems. Once each supramolecular complex was formed in the solubilized phase of the aqueous solution, the effective inclusion of the drugs and the stoichiometric relationship of both systems were analyzed using ^1^H-NMR.

**Figure 4:**
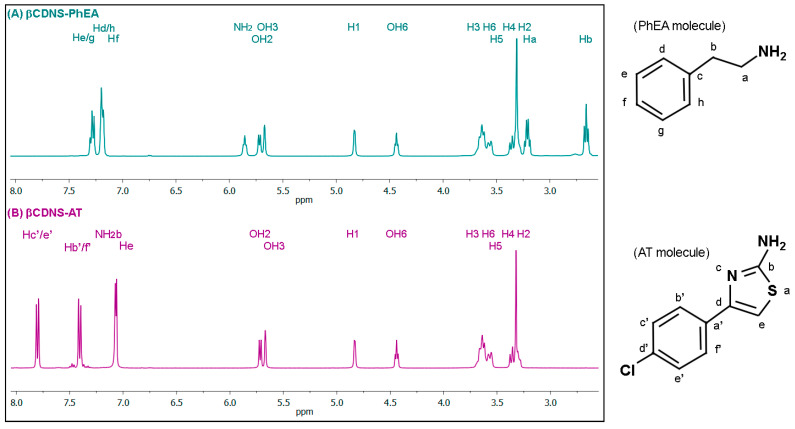
^1^H-NMR spectra of (**A**) phenylethylamine (PhEA) loaded in βCDNS (βCDNS–PhEA) and (**B**) 2-amino-4-(4-chlorophenyl)-thiazole (AT) loaded in βCDNS (βCDNS–AT) (**left**) together with the molecular structures and the assigning protons with respect to PhEA and AT (**right**).

**Figure A4:**
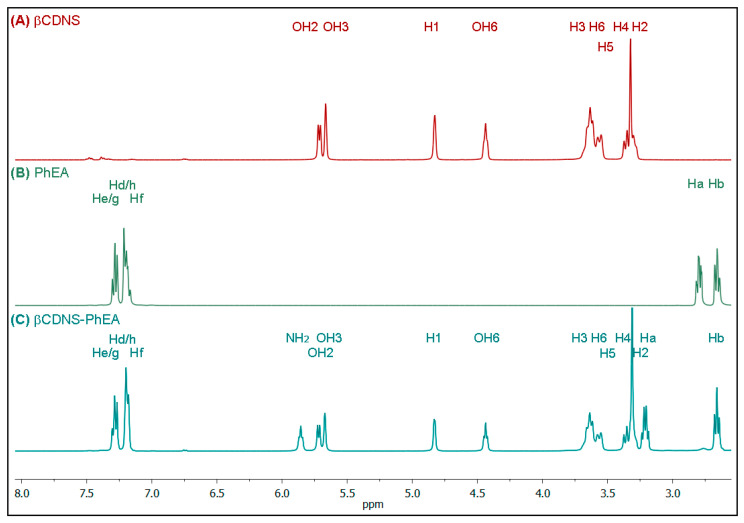
^1^H-NMR spectra of (**A**) βCDNS, (**B**) PhEA and (**C**) βCDNS-PhEA.

**Figure A5:**
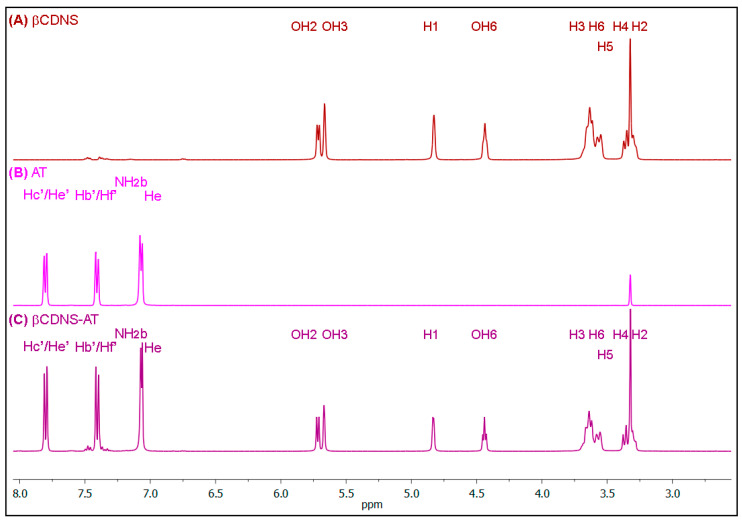
^1^H-NMR spectra of (**A**) βCDNS (**B**) AT and (**C**) βCDNS-AT.

Figure 4 shows the spectra of βCDNS–PhEA (A) and βCDNS–AT (B) with their molecular structures and proton assignments for the respective drug. The loading of PhEA to form the βCDNS–PhEA system (A) and the loading of AT to form the βCDNS–AT system (B) were confirmed with the respective assignments of protons in the molecular structures of PhEA and AT (see full spectra, Figure A4 and Figure A5, in Appendix B). Table 2 and Table 3 show the chemical shifts and integrals recorded for the protons of βCDNS and of the PhEA and AT drugs resulting from the inclusion process.

For the βCDNS–PhEA system, Table 2, the largest chemical shifts for βCDNS were observed for the internal protons H3 and H5 and the hydroxyl groups OH2 and OH3, probably due to preferential inclusion in the widest zone of the βCD cavity. In addition, chemical shifts for all the βCDNS protons were observed, mainly towards lower fields, which demonstrates the effective loading of PhEA within βCD cavities and in the multiple interstitial spaces of the interstitial βCDNS produced by crosslinking. Analyzing the chemical shifts of the PhEA protons, a change in the chemical environment due to inclusion was also evidenced, consistent with that reported in the literature [25,71].

For the βCDNS–AT system, Table 3, chemical shifts were observed in all the βCDNS protons oriented towards the interior and exterior of the cavity due to the change in the chemical environment of βCDNS resulting from AT loading. This finding shows that the inclusion of the drug occurs in βCD cavities and between the formed interstitial spaces. Chemical shifts towards higher fields were observed in the protons NH_2_b, Hb’/f’, and Hc’/e’ of AT, which demonstrates the electronic shielding effect of the drug due to its inclusion in the nanosponges, in accordance with that reported in the literature [26].

Notably, the integration of the βCDNS protons and the protons of each drug in their respective ^1^H-NMR spectra, Table 2 and Table 3, showed a stoichiometric βCD:drug ratio of 1:8 in both systems, which is an amount of drug eight times greater than those reported for βCD–PhEA [25] and βCD–AT [26], each of which exhibits a 1:1 stoichiometry. This amount is equivalent to 0.9 mg of PhEA loading per 1 mg of βCD unit in βCDNS, and on the other hand, to 1.5 mg of AT loading per 1 mg of βCD unit in βCDNS. Applying Equation (1) [58] (Section 2, Material and Methods), the loading capacity in βCDNS is 90% for PhEA and 150% for AT, which is higher than the loading capacity of 11% for PhEA and 19% for AT in βCD native, according to reported data [25,26]. These results show that the drug loading of the βCDNS formed is higher than that of native βCD and that βCDNS could be used as a more efficient drug carrier than native βCD (see the details in the Appendix B).

**Figure 5:**
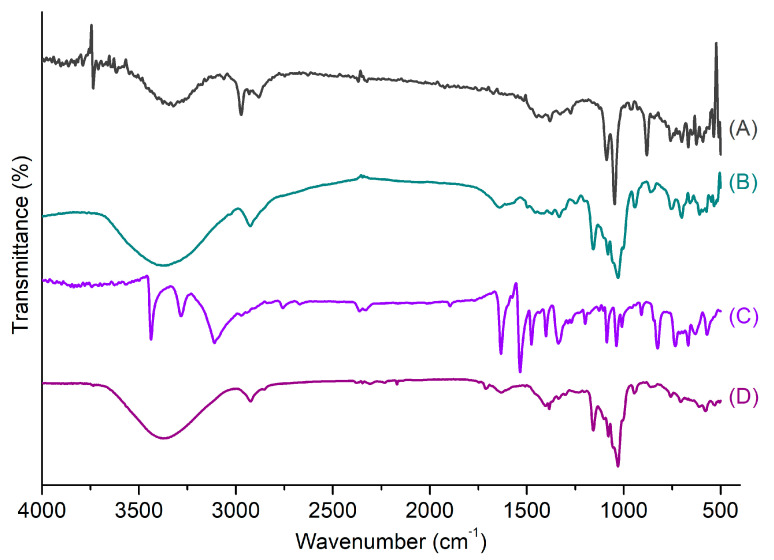
IR spectra of (**A**) phenylethylamine (PhEA), (**B**) PhEA loaded in βCDNS (βCDNS–PhEA), (**C**) 2-amino-4-(4-chlorophenyl)-thiazole (AT), and (**D**) AT loaded in βCDNS–AT.

The loading of drugs into βCDNS was also analyzed by FT-IR spectroscopy by comparing peaks for vibrations before and after the inclusion process. Figure 5 shows the spectra of (A) PhEA, (B) βCDNS-PhEA, (C) AT, and (D) βCDNS–AT.

In the vibrational analysis of the βCDNS–PhEA system, the βCDNS peaks at 3570 cm^−1^ and 3170 cm^−1^ corresponding to O-H alcohol stretching and N-H primary amine asymmetric and symmetric stretching, respectively, were identified. The peaks at 2926 cm^−1^ corresponding to C-H stretching, at 1642 cm^−1^ corresponding to C=O stretching, at 1333 cm^−1^ and 1157 cm^−1^ corresponding to O-H bending, and at 1081 cm^−1^ and 1029 cm^−1^ corresponding to C-O stretching were also identified. These vibrations remain unchanged in comparison to those of the βCDNS spectrum without loaded drugs. The peak from PhEA found for the βCDNS–PhEA system corresponding to N-H symmetric stretching was observed at 2950 cm^−1^, while the peak at 745 cm^−1^ corresponding to C-H aromatics was masked due to the inclusion process.

In the case of the βCDNS–AT system, decreases in the intensity of some peaks with respect to those of βCDNS were observed. However, the characteristic peaks were located in the same regions of the spectra. O-H alcohol stretching, and N-H primary amine asymmetric and symmetric stretching vibrations were observed at 3170 cm^−1^ and 3570 cm^−1^, respectively. C-H stretching appeared at 2924 cm^−1^, C=O stretching at 1637 cm^−1^, O-H group bending at 1384 cm^−1^ and 1157 cm^−1^, and finally, C-O stretching appeared at 1079 cm^−1^ and 1029 cm^−1^. The characteristic peaks of AT at 1476 cm^−1^, corresponding to C=C aromatics, and at 3438 cm^−1^, corresponding to N-H aromatic stretching, were masked in βCDNS–AT due to the inclusion in βCDNS.

**Figure 6:**
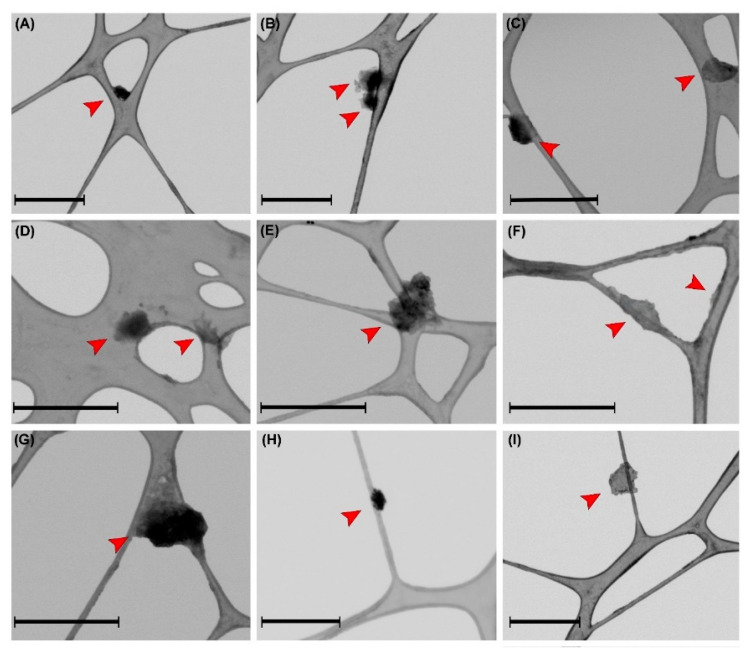
TEM micrographs of (**A**–**E**) βCDNS–PhEA and (**F**–**I**) βCDNS–AT. Scale bar for all images is 1000 nm (Red arrows highlight the nanosystems in the micrographs).

The changes in the intensity and definition of the βCDNS peaks observed in the IR spectra suggested a change in their conformations due to drug loading, which was also corroborated by DLS and TEM. The hydrodynamic diameters of βCDNS–PhEA and βCDNS–AT were 270.5 ± 48.0 nm and 335.5 ± 150.5 nm, respectively, observing an increase in the size of both systems with respect to βCDNS (see more details in Appendix C). Figure 6 shows TEM images of βCDNS loaded with PhEA (A–E) and AT (F–I). Changes in the shapes of the systems with respect to that of βCDNS were also observed; in addition, the average diameter calculated using TEM images of these systems increased to 252 ± 39 nm with respect to βCDNS. The loading of the drugs PhEA and AT could promote a process of association and intermolecular interactions between different βCDNS. This would explain the increase in size observed using TEM and DLS.

The degree of solubilization indicates the tendency to increase the aqueous solubility of the drugs due to the action of βCDNS, while the complexation efficiency corresponds to the concentration of drug included versus the concentration of drug initially used in the process. This is directly related to the effectiveness of βCDNS and intermolecular interactions to keep drugs entrapped in the complex. The degree of solubilization of the drugs, the *K_a_*, and the complexation efficiency for the βCDNS–PhEA and βCDNS–AT systems were calculated using phase solubility studies (Equations (2) and (3), Section 2, Material and Methods) [59] and are shown in Table 4. Additionally, they were compared with the results obtained for the complexation of PhEA and AT using native βCD [25,26].

**Figure A7:**
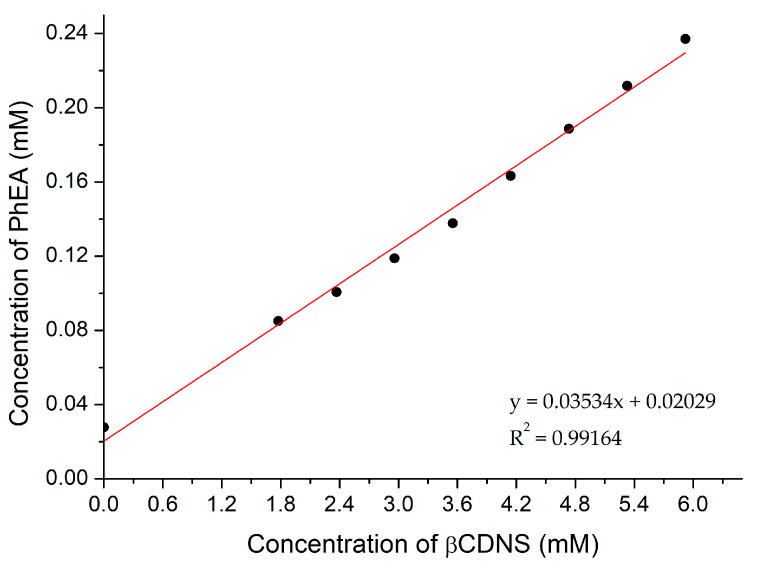
Graph of the concentration of solubilized PhEA versus the concentration of added βCDNS and the linear fit.

**Figure A8:**
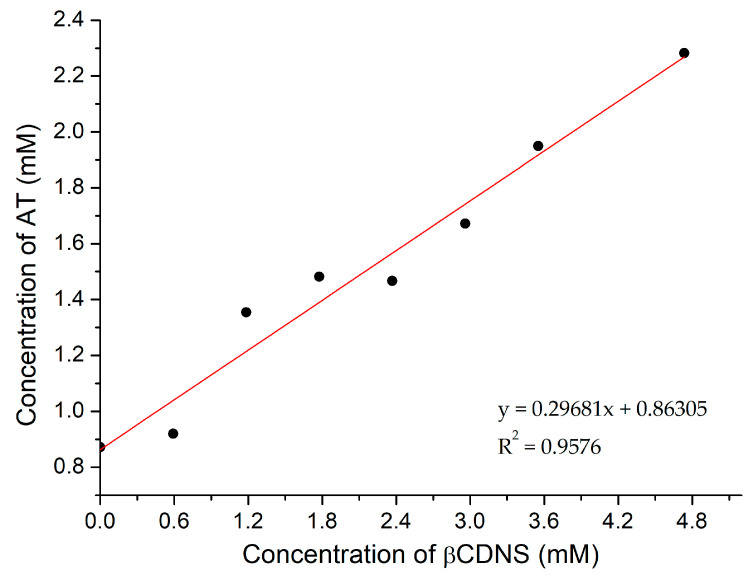
Graph of the concentration of solubilized AT versus the concentration of added βCDNS and the linear fit.

An increase in the aqueous solubility of PhEA and AT using βCDNS was observed, when they were compared to the solubility of free drugs (see Figure A7 and Figure A8, Appendix B). Notably, the degree of solubilization achieved by the presence of βCDNS was more than 1.3 times higher for PhEA and 5 times higher for AT than with native βCD. This is especially relevant in therapy since drugs to be pharmacologically active must be soluble in water. The *K_a_* values are 1318 M^−1^ and 484 M^−1^ for the βCDNS–PhEA and βCDNS–AT systems, respectively. These results indicate that the interactions that allow inclusion are strong, forming two highly stable systems over time due to the incorporation of βCDNS. The complexation efficiency values obtained for both systems show that the complexation using βCDNS is optimal, being the same for PhEA in native βCD and seven times greater for AT in native βCD. The above findings are in accordance with the previous discussion given by stoichiometry studies and loading capacity calculated using NMR (more details in the Appendix B).

In general, the K_a_ values of the βCD complexes vary between 50 and 2000 M^−1^. Lower values at 50 M^−1^ indicate a limitation in the pharmaceutical formulation since they have low stability and do not release the drug at its site of action [25,72,73,74]. On the other hand, K_a_ values greater than 2000 M^−1^ also present limitations, such as poor pharmacokinetics, since the drug release rates can be affected [72,73]. This is why the use of a strategy for the controlled release of the drugs included in βCDNS becomes relevant. AuNPs can release absorbed energy in the form of heat and can release molecules near their surface as a result of the photothermal effect [28,75,76]. This was demonstrated for a drug in AuNP- and βCD-based systems using laser irradiation [25,47]. In this sense, the incorporation of AuNPs into the two systems could, in addition to acting as a therapeutic agent, promote the controlled release of the drugs.

### 3.3. Synthesis and Immobilization of Gold Nanoparticles on Drug-Loaded β-Cyclodextrin-Based Nanosponges

**Figure 7:**
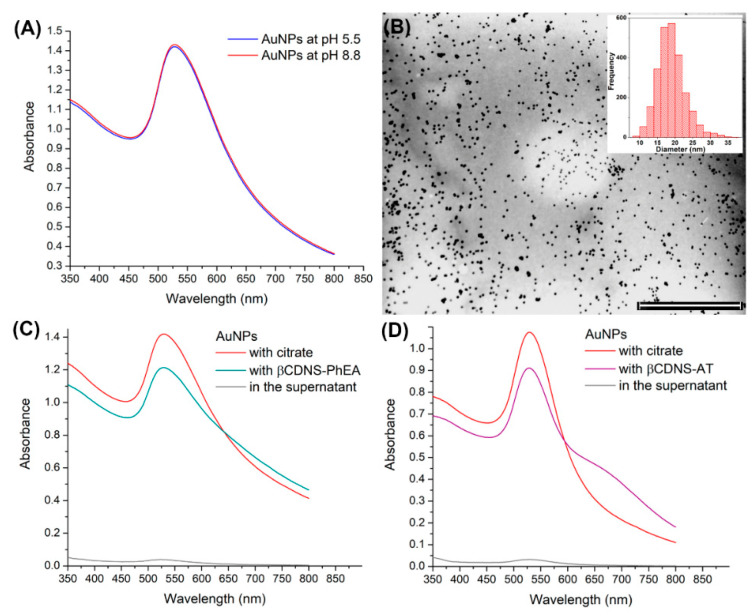
(**A**) UV-Vis spectra of AuNPs at pH 5.5 and pH 8.8; (**B**) TEM micrograph of AuNPs with their size histogram inserted (scale bar of 1000 nm); (**C**) UV-Vis spectra of AuNPs with citrate and with βCDNS–PhEA, including supernatant of the functionalization; and (**D**) UV-Vis spectra of AuNPs with citrate and with βCDNS–AT, including supernatant of the functionalization.

**Figure A9:**
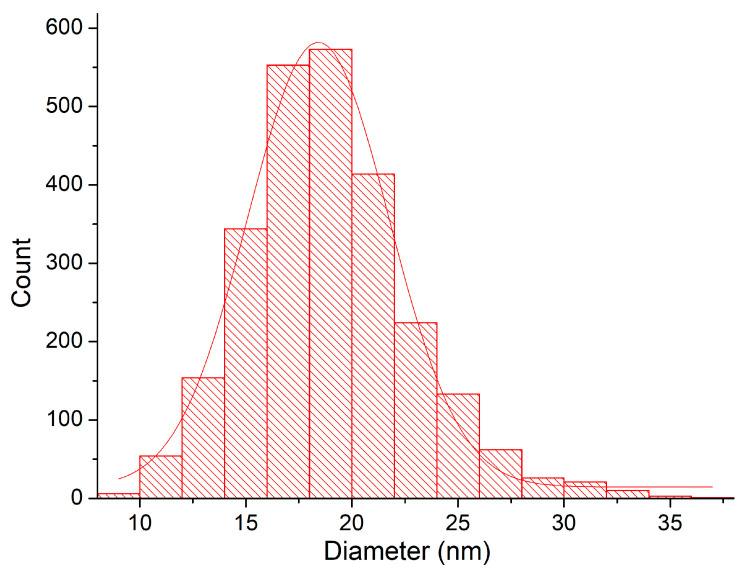
Histogram of size distribution of synthesised AuNPs.

Once the βCDNS–drug systems were obtained, the interactions with colloidal AuNPs were studied to load another therapeutic agent and form the βCDNS–PhEA–AuNP and βCDNS–AT–AuNP systems. AuNPs were synthesized following the Turkevich method at pH 5.5. These AuNPs were then stabilized at pH 8.8 to facilitate their immobilization on drug-loaded βCDNS. Figure 7A shows the absorbance spectra of AuNPs at pH 5.5 and 8.8, and Figure 7B shows a representative TEM micrograph of spherical AuNPs with an average diameter of 18 ± 4 nm (see histogram in Figure A9, Appendix C) AuNPs with diameters between 4 and 100 nm do not present cytotoxic effects [77], which would allow possible drug delivery applications.

Figure 7C,D shows the UV-Vis spectra of the βCDNS–PhEA–AuNP and βCD–AT–AuNP systems, respectively, in addition to those of the initial AuNP solution and the supernatant resulting from the functionalization of each mixture. The recorded plasmon bands demonstrate a preferential interaction of AuNPs with βCDNS–drug, with an immobilization of 85%, maintaining the main characteristics of the plasmon band and indicating that AuNPs remain stable in both systems.

Table 5 shows the intensities and the maximum wavelengths from the absorbance spectra. In addition, the hydrodynamic diameter and surface charge of βCDNS–PhEA–AuNP and βCDNS–AT–AuNP in aqueous solution are shown. These analyses represent the behavior of AuNPs in the different systems, because Au is highly efficient to absorb and scatter light, being superior to the organic material present.

A shift in the wavelength of the maximum absorbances with respect to those for the as-synthesized AuNPs occurred for both systems due to the interparticle coupling caused by the increased proximity between these nanostructures when immobilized; in turn, the permanence of the plasmon bands was evidence of the stability achieved and that the aggregation of AuNPs did not occur. In turn, increases in hydrodynamic diameters from 33.9 ± 13.2 nm for AuNPs with citrate to 51.2 ± 24.7 nm for AuNPs in the βCDNS–PhEA–AuNP system and up to 114.0 ± 42.2 nm for AuNPs in the βCDNS–AT system were observed due to the proximity between the immobilized AuNPs and the presence of βCDNS–drug complexes. Furthermore, this behavior was consistent with the increase in size of the βCDNS when they were loaded with the drugs. The reported partial and dynamic inclusion of AT in βCD could explain the greater hydrodynamic diameter of the AuNPs on βCDNS–AT with respect to βCDNS–PhEA. The two functional groups, NH_2_ and SH, of AT are exposed [26], facilitating its interaction with AuNPs, while PhEA only has one NH_2_ group that is completely included within βCD [25,71].

The registered surface charge of the AuNPs was −51.4 ± 7.9 mV due to the stabilizing citrate ions, which changed to −33.0 ± 5.3 mV and −38.4 ± 6.9 mV for AuNPs in the βCDNS–PhEA–AuNP and in the βCDNS–AT–AuNP systems, respectively, due to the replacement of a fraction of citrate molecules by neutral supramolecular complexes. As a control, a drug-free βCDNS solution was subjected to the same mixing protocol with colloidal AuNPs, confirming through different characterization techniques that the interaction between βCDNS and AuNPs does not occur (see the details in the Appendix C).

**Figure 8:**
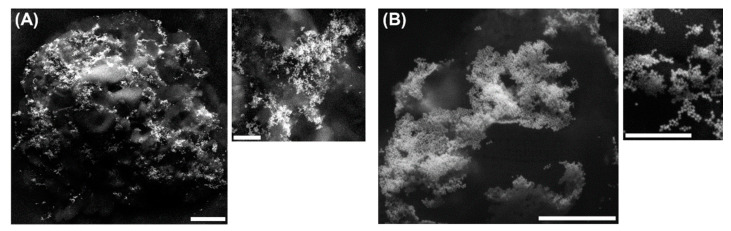
SEM micrographs of (**A**) βCDNS–PhEA with gold nanoparticles (AuNPs) immobilized on the surface and a zoomed view, with bar scales of 2000 nm and 500 nm; (**B**) βCDNS–AT with AuNPs immobilized on its surface and a zoomed view, with bar scales of 1000 nm and 500 nm.

Figure 8A,B shows SEM micrographs of the βCDNS–PhEA–AuNP (A) and βCDNS–AT–AuNP (B) systems, respectively. The images clearly show the AuNPs immobilized on the βCDNS–drug supramolecular complexes. In addition, an irregular morphology was observed, probably due to the process of functionalization of βCDNS, as suggested by the TEM images (Figure 6).

Various characterization techniques and direct observation using electron microscopy confirmed the simultaneous loading of βCDNS with two therapeutic agents, drugs and AuNPs, forming the βCDNS–PhEA–AuNP and βCDNS–AT–AuNP systems. If properly designed, that is, by establishing parameters for the colloidal stability, concentration, surface charge, and size, among others, βCDNS and AuNPs could be considered nontoxic and used in therapy without generating adverse effects in the organism. In this sense, in the design and formation of these two new systems, the established parameters were realized.

## 4. Conclusions

The formation of βCDNS was confirmed by different techniques that indicated its polymeric characteristics and nanometric dimensions. Therapeutic agents PhEA and AT were successfully included in the multiple cavities of the nanostructures, forming the βCDNS–PhEA and βCDNS–AT systems. The loading capacity of βCDNS was 90% for PhEA and 150% for AT, being eight times higher than with native βCD. An increase in the aqueous solubility of PhEA and AT when complexed with βCDNS was demonstrated. In addition, a higher degree of solubilization and complexation efficiency of both drugs was obtained with βCDNS than with native βCD. The synthesized AuNPs were also loaded into each system, reaching an immobilization percentage of 85%. The hydrodynamic diameter and surface charge of AuNPs were 51 nm and −33 mV in the βCDNS–PhEA–AuNP system and 114 nm and −38 mV in the βCDNS–AT–AuNP system, respectively, which are relevant parameters for biological studies. βCDNS loaded with the two therapeutic agents (drug and AuNP) were observed directly by SEM images, showing the porous morphologies of the nanosponges and the nanoparticles immobilized on their surfaces due to the SH and NH_2_ functional groups of the drugs. We believe that these unique systems, based on βCDNS, drugs, and AuNPs, can be versatile materials with an efficient loading capacity for potential applications in the transport of therapeutic agents. Finally, to continue researching in the field of drug delivery, studies that demonstrate the controlled release of PhEA and AT from βCDNS–drug–AuNP using laser irradiation are required and this, together with studies of cell permeability, toxicity, and pharmacological activity, has been considered in a future perspective.

## Figures and Tables

**Table 1:** Proton assignments, ^1^H-NMR chemical shifts, and integrations of the β-cyclodextrin (βCD) and β-cyclodextrin-based nanosponge (βCDNS) signals.

Protons	δβCD (ppm)	δβCDNS (ppm)	|Δδ| (ppm)	βCD Integration (∫)	βCDNS Integration (∫)	|Δ∫|
H1	4.825	4.827	0.002	7.00	7.00	0
H2	3.311	3.301	0.010	6.99	6.98	0.01
H3	3.669	3.655	0.014	6.99	6.98	0.01
H4	3.370	3.361	0.009	7.14	7.15	0.01
H5	3.566	3.562	0.004	7.04	7.04	0
H6	3.621	3.624	0.003	13.83	13.84	0.01
OH2	5.706	5.714	0.008	7.01	6.68	0.33
OH3	5.661	5.665	0.004	7.02	6.57	0.45
OH6	4.435	4.437	0.002	7.15	6.43	0.72

**Table 2:** Proton assignments, ^1^H-NMR chemical shifts, and integrations of the βCDNS, phenylethylamine (PhEA), and PhEA loaded in βCDNS (βCDNS–PhEA) signals.

**Proton**	**δβCDNS (ppm)**	**δβCDNS–** **PhEA (ppm)**	**|Δδ| (ppm)**	**βCDNS Integration (** ** ∫ ** **)**	**βCDNS–** **PhEA Integration (** ** ∫ ** **)**
H1	4.827	4.829	0.002	7.00	7.00
H2	3.301	-	-	6.98	-
H3	3.655	3.660	0.005	6.98	6.96
H4	3.361	3.363	0.002	7.15	7.03
H5	3.562	3.565	0.003	7.04	7.05
H6	3.624	3.626	0.002	13.84	13.93
OH2	5.713	5.719	0.006	6.68	6.67
OH3	5.665	5.671	0.006	6.57	6.64
OH6	4.437	4.436	0.001	6.43	6.51
**Proton**	**δPhEA (ppm)**	**δβCDNS–** **PhEA (ppm)**	**|Δδ| (ppm)**	**PhEA** **Integration (** ** ∫ ** **)**	**βCDNS–** **PhEA Integration (** ** ∫ ** **)**
NH_2_	1.385	5.856	4.471	2.05	7.07
Ha	2.798	3.211	0.413	2.05	15.11
Hb	2.658	2.660	0.002	2.07	16.05
Hd/h	7.205	7.198	0.007	1.97	16.00
He/g	7.285	7.284	0.001	2.00	16.02
Hf	7.172	7.179	0.007	0.96	7.70

**Table 3:** Proton assignments, ^1^H-NMR chemical shifts, and integrations of the βCDNS, 2-amino-4-(4-chlorophenyl)-thiazole (AT) and AT loaded in βCDNS (βCDNS–AT) signals.

**Proton**	**δβCDNS (ppm)**	**δβCDNS–AT (ppm)**	**|Δδ|(ppm)**	**βCDNS Integration (** **∫)**	**βCDNS–AT Integration (** **∫)**
H1	4.827	4.832	0.005	7.00	7.00
H2	3.301	-	-	6.98	-
H3	3.655	3.664	0.009	6.98	7.01
H4	3.361	3.367	0.006	7.15	7.09
H5	3.562	3.568	0.006	7.04	7.04
H6	3.624	3.629	0.005	13.84	13.86
OH2	5.713	5.717	0.004	6.68	6.70
OH3	5.665	5.669	0.004	6.57	6.53
OH6	4.437	4.440	0.003	6.43	6.53
**Proton**	**δAT (ppm)**	**δβCDNS–AT (ppm)**	**|Δδ| (ppm)**	**AT Integration (** **∫)**	**βCDNS–AT Integration (** **∫)**
He	7.061	7.061	0.000	0.99	8.49
NH2 b	7.079	7.073	0.006	2.00	16.10
Hb’/f’	7.408	7.407	0.001	1.95	16.09
Hc’/e’	7.803	7.801	0.002	2.00	16.52

**Table 4:** Comparative data on the degree of solubilization, association constants, and complexation efficiency of the drugs PhEA and AT included in βCDNS versus native βCD.

System	Degree of Solubilization	K_a_ (M^−1^)	Complexation Efficiency
βCDNS–PhEA	0.035	1318	0.037
βCDNS–AT	0.297	484	0.422
βCD–PhEA *	0.028	760	0.038
βCD–AT *	0.051	970	0.054

* Reference values obtained from the literature [25,26].

**Table 5:** Data obtained from the UV-VIS spectra, dynamic light scattering (DLS), and ζ potentials of the as-synthesized gold nanoparticles (AuNPs) and AuNPs immobilized on the βCDNS–PhEA and βCDNS–AT supramolecular systems.

System	Intensity (a.u.) of A_max_	Wavelength (nm) of A_max_	Immobilized AuNPs (%)	Hydrodynamic Diameter (nm)	PDI	Superficial Charge (mV)
AuNPs–citrate	1.419	528	0	33.9 ± 13.2	0.537	−51.4 ± 7.9
βCDNS–PhEA–AuNP	1.214	529	85.5	51.2 ± 24.7	0.571	−33.0 ± 5.3
βCDNS–AT–AuNP	0.912	531	84.7	114.0 ± 42.2	0.663	−38.4 ± 6.9

**Figure A6:**
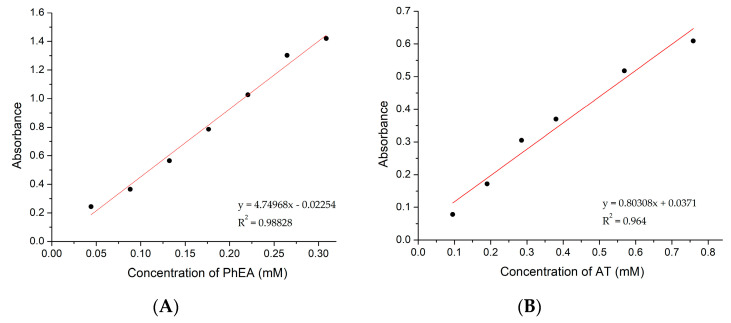
Linear plots of the (**A**) PhEA and (**B**) AT absorbance maxima at 310 and 290 nm, respectively, vs. concentrations.

## Data Availability

Not applicable.

## Appendix A

### Appendix A.1. β-cyclodextrin Nanosponge Synthesis

The methods for the synthesis of βCDNS were:

#### Appendix A.1.1. Method A. (Patel, 2014)

In a round-bottomed flask, βCD was mixed with DPC, and two different molar ratios were studied: 1:4 and 1:8. The flask was then placed in an ultrasonic bath at 90 °C for 5 h. The solid product obtained was then repeatedly washed with distilled water and vacuum filtered for 2 h using a Kitasato flask with a Büchner funnel with filter paper. The product was washed for 24 h in a Soxhlet system, and two different solvents, ethanol and acetone, were studied for this process; the product was finally dried and stored in an amber flask with a Teflon seal.

#### Appendix A.1.2. Method B. (Modified from Patel)

In a round-bottomed flask, βCD was mixed with DPC, and two different molar ratios were studied: 1:4 and 1:8. The flask was heated in an oil bath on a heating plate with constant agitation for 5 h at 100 °C. The mouth of the flask was covered with a septum, and a syringe was introduced into it to let the phenol gas released from the reaction pass through. The solid mixture obtained was extracted from the flask, a small amount of water was added and an ultrasonic bath was used to release residues from the walls. Once all the solid was extracted, it was ground in a mortar, washed with distilled water, and then vacuum filtered for 2 h. The product was washed for 24 h, and two different solvents, ethanol and acetone, were studied for this process: ethanol and acetone. Finally, the product was dried for between 48 and 72 h at 65 °C and stored.

Figure A1 shows a bar graph with the mass yields of different types of βCDNS syntheses with changes in the heating method, the solvents used in the washing steps and the βCD:DPC molar ratio.

### Appendix A.2. Derivative Curves from TGA Characterization of β-Cyclodextrin Nanosponge

Figure A2 shows the derivative curves from TGA (Figure 2B), analysing the differences in the peaks of the thermogravimetric decomposition curves.

### Appendix A.3. TEM Characterization of Native β-Cyclodextrin

Figure A3 shows a TEM image of Figure A3A–C βCDNS to obtain its mean size; and Figure A3D native βCD with the same dispersion and staining protocol described for βCDNS.

## Appendix B

### Appendix B.1. ^1^H-NMR Full Spectra of Loading Drugs Process

Figure A4 and Figure A5 show the full ^1^H-NMR spectra of βCDNS, PhEA, AT, βCDNS-PhEA and βCDNS-AT, summarized in Figure 4.

### Appendix B.2. Calculation of the Stoichiometric Ratio of the Drug Loading Process

The stoichiometric ratios were calculated in the ^1^H-NMR spectra by comparing the integrals of the PhEA and AT protons with the integrals of the βCDNS protons from the βCDNS-PhEA and βCDNS-AT systems. First, the integrals of the PhEA signals (protons He/g, Hd/h, and Hf) were analysed using the H1 signal of βCDNS as reference, which integrated 7. In turn, the integrals of the AT signals (He, Hb’/f’, Hc’/e’ and NH_2_b) were analysed using the H1 signal of βCDNS as reference, which integrated 7 (see the data in Table A1 and Table A2). Finally, the stoichiometric ratios calculated are summarized in Table A3.

**Table A1:** Values of the integrated PhEA and βCD proton signals in the ^1^H-NMR spectra of the βCDNS-PhEA system, with the integrated H1 proton signals of βCDNS as a reference.

Proton Signal	Reference	Integral	Counts	Ratios
H1	7	7.0	1	1
He/g	2	16.0	8.0	8
Hd/h	2	16.0	8.0	8
Hf	1	7.7	7.7	8

**Table A2:** Values of the integrated AT and βCD proton signals in the ^1^H-NMR spectra of the βCDNS-AT system, with the integrated H1 proton signals of βCDNS as a reference.

Proton Signal	Reference	Integral	Counts	Ratios
H1	7	7	1	1
He	1	8.5	8.5	8
Hb’/f’	2	16.1	8.1	8
Hc’/e’	2	16.1	8.1	8
NH_2_b	2	16.5	8.3	8

**Table A3:** Summary of the molar ratios of the drugs in the βCDNS for βCDNS-drug systems calculated from Table A1 and Table A2.

Molar Ratios		
System	βCDNS	Drug
βCDNS-PhEA	1	8 (±0.3)
βCDNS-AT	1	8 (±0.4)

### Appendix B.3. Extinction Coefficient Determination of Drugs

For each drug, a calibration curve was made with stocks of aqueous solutions of known concentrations to obtain the value of ε. Table A4 presents the data used for this determination for PhEA and AT.

By plotting the PhEA concentration versus the maximum absorbance at 310 nm, the line shown in Figure A6A was obtained, and the value of ε was 4.7497 ± 0.2110 mM^−1^cm^−1^. By plotting the AT concentration versus the maximum absorbance at 290 nm, the line presented in Figure A6B was obtained; the slope corresponds to the value of ε, which was 0.8031 ± 0.0692 mM^−1^cm^−1^.

**Table A4:** Data on the concentrations of the drugs, PhEA and AT, and their absorbance maxima at 310 and 290 nm, respectively.

[PhEA] (mM)	Absorbance	[AT] (mM)	Absorbance
0.0441	0.2441	0.095	0.078
0.0882	0.3648	0.190	0.172
0.1323	0.5642	0.285	0.305
0.1764	0.7851	0.380	0.370
0.2205	1.0267	0.569	0.518
0.2646	1.3023	0.759	0.609
0.3087	1.4199	---	---

### Appendix B.4. Determinations of Association Constants of Drug Loading Systems

For the determination of *K_a_*, a stock solution was prepared with 200 mg of βCDNS and water in a 25 mL measuring flask. Volumes of 0 to 2 mL of the stock were taken and diluted with water to produce a total volume of 2 mL with a fixed amount of each drug, 0.5 mL for PhEA and 5.0 mg for AT, added. All the data obtained are presented in Table A5 for PhEA and Table A6 for AT. By applying the extinction coefficient value ε to the Lambert-Beer equation, it was possible to determine the PhEA and AT concentrations in the different assays using the Higuchi-Connors method.

**Table A5:** Values of the different tests carried out to calculate the K_a_ and complexation efficiency of the βCDNS-PhEA system in water.

[βCDNS] (mM)	Absorbance	[PhEA] (mM)
5.920	1.126	0.237
5.328	1.006	0.212
4.736	0.896	0.189
4.144	0.775	0.163
3.552	0.654	0.138
2.960	0.564	0.119
2.368	0.478	0.101
1.776	0.404	0.085
0	0.132	0.028

**Table A6:** Values of the different tests carried out to calculate the K_a_ and complexation efficiency of the βCDNS-AT system in water.

[βCDNS] (mM)	Absorbance	[AT] (mM)
4.736	1.833	2.282
3.552	1.566	1.950
2.960	1.343	1.672
2.368	1.178	1.467
1.776	1.19	1.482
1.184	1.088	1.355
0.592	0.739	0.920
0	0.7	0.872

The linear relationship obtained from a plot of the solubilized PhEA concentration versus the added βCDNS concentration is shown in Figure A7. The value of the slope was 0.03534 (±0.00115). Using Equation (1), the association constant K_a_ was calculated, resulting in a value of 1318 M^−1^. Finally, using Equation (2), the value of complexation efficiency was calculated, resulting in a value of 0.03663 for the βCDNS-PhEA system.

Figure A8 shows the linear relationship obtained from a plot of the solubilized AT concentrations versus the added βCDNS concentration. The value of the slope was 0.297 (±0.024). The K_a_ for the βCDNS-AT system was 484 M^−1^ and the complexation efficiency value was 0.422.

## Appendix C

### Appendix C.1. Size Histogram of Gold Nanoparticles

Figure A9 shows the size distribution histogram for synthesised AuNPs from representative TEM images. The observed diameter was 18 (±4) nm.

### Appendix C.2. Dynamic Light Scattering and ζ Potential Studies of Loading Systems and Gold Nanoparticles Interacting with Supramolecular Systems

The studies using DLS and the ζ potential are summarized in Table A7. βCDNS was dispersed in water (pH 8.8) and measured, while βCDNS-drug systems were sonicated and measured. AuNPs stabilized with citrate were filtered and then characterized. AuNPs with βCDNS and drugs were centrifuged and resuspended in water (pH 8.8) prior to characterization.

**Table A7:** Data obtained using dynamic light scattering (DLS) and the ζ potential of βCDNS and βCDNS-drugs, and AuNPs with citrate, βCDNS, βCDNS-PhEA and βCDNS-AT.

System	Hydrodynamic Diameter (nm)	Intensity Peak Area (%)	PDI	Surface Charge (mV)
βCDNS	133.9 ± 66.9	100.0	0.197	
βCDNS-PhEA	270.5 ± 48.0	87.0	0.398	
βCDNS-AT	335.5 ± 150.5	89.0	0.355	
AuNPs-citrate	33.9 ± 13.2	69.7	0.537	−51.40 ± 7.86
AuNPs-βCDNS	34.6 ± 12.9	70.0	0.542	−58.03 ± 7.01
βCDNS-PhEA-AuNP	51.2 ± 24.7	85.8	0.571	−33.03 ± 5.26
βCDNS-AT-AuNP	114.0 ± 42.2	86.3	0.663	−38.37 ± 6.90
